# The Third Volume

**Published:** 1857-09

**Authors:** 


### EDITORIAL AND MISCELLANEOUS.

### The Third Volume.

At the commencement of the Third Volume of our Journal, we must be permitted to express our gratification at the position which it occupies, as evinced in the most unequivocal manner from various sources. We do not refer to this matter with any view of boasting or lauding our own labors—it is true, however, that a large amount of time and labor which, in a pecuniary point of view, might have been more profitably employed, has been expended upon it—but the object is to express our gratitude to those who have appreciated our labors, and encouraged us continually by kind words, and it is especially an object to make the acknowledgement of obligations due our collaborators, who have of course mainly contributed to the establishment of the Journal upon its present gratifying basis.

In this connection, we would remark that though, as stated in the last number of our Journal, it has changed hands, (so far as its *publication* is concerned, the *editorial* connection remaining the same,) it is with no diminished interest in its success that we again present its claims to the profession. We are assured that at present it exhibits regularly not only a large amount of valuable original matter, from the medical men of the South, a considerable portion of which will compare favorably with the contributions of any Journal in the United States; but we have no hesitation in asserting that with this Journal alone, medical men would not be kept in ignorance of the medical progress of the day; our object being (in which we think there has not been an entire failure) to present a synopsis of medical advance.

Having no direct pecuniary interest in the publication, we have less hesitation in urging our friends, and the friends of Southern medicine particularly, to increase their exertions to extend its circulation, assuring them that in proportion to the patronage and support given, will be the effort to increase its interest and value.

